# Neutrophil CD64, CD66b, and Serum Thioredoxin as Early Biomarkers of Delayed Fracture-Related Infection After Internal Fixation: A Prospective Cohort Study

**DOI:** 10.7759/cureus.108619

**Published:** 2026-05-10

**Authors:** Sabir Ali, Salma Siddiqui, Ajai Singh, Manish Yadav, Aman Ali, Rehan Ul Haq, Abbas A Mehdi

**Affiliations:** 1 Multidisciplinary Research Unit, All India Institute of Medical Sciences, Bhopal, Bhopal, IND; 2 Pediatric Orthopedics, King George's Medical University, Lucknow, IND; 3 Health Research, All India Institute of Medical Sciences, Bhopal, Bhopal, IND; 4 Statistics, University of Lucknow, Lucknow, IND; 5 Orthopedics, All India Institute of Medical Sciences, Bhopal, Bhopal, IND; 6 Biochemistry, Era's Lucknow Medical College and Hospital, Era University, Lucknow, IND

**Keywords:** antigens, biomarkers, bone, cd, fracture fixation, fractures, internal, surgical wound infection, thioredoxins

## Abstract

Background: Delayed fracture-related infection (FRI) remains a major complication after internal fixation, and conventional inflammatory markers have limited diagnostic accuracy. This study evaluated neutrophil CD64, CD66b, and serum thioredoxin as adjunctive biomarkers for delayed FRI.

Methods: In this prospective cohort study at a tertiary trauma center, 637 patients undergoing internal fixation for long-bone fractures were enrolled and followed until fracture union. Venous blood was collected pre-operatively and on postoperative days 3, 7, and 10. Total leucocyte count (TLC), erythrocyte sedimentation rate (ESR), C-reactive protein (CRP), neutrophil CD64 and CD66b, and serum thioredoxin were measured. FRI was defined using contemporary clinicoradiological and microbiological consensus criteria. Diagnostic performance was assessed by receiver operating characteristic (ROC) analysis.

Results: Delayed FRI occurred in 56/637 patients (8.8%). FRI was associated with open fractures (30.4% vs 9.0%) and Tscherne grade ≥2 (78.6% vs 61.1%) and significantly prolonged time to union (21.84 ± 6.92 vs 16.26 ± 3.42 weeks; p < 0.0001). From day 3 onward, TLC, ESR, and CRP were significantly higher in the FRI group; CRP on day 7 showed the best conventional performance (cut-off >20 mg/L; sensitivity 83%, specificity 80%, AUC 0.85). CD64, CD66b, and thioredoxin diverged sharply between groups. CD64 on day 10 (cut-off >3800 median fluorescence intensity (MFI)) yielded a sensitivity of 91%, a specificity of 87%, NPV of 99% (AUC 0.93). CD66b on day 3 (>450 MFI) and thioredoxin on day 7 (>85 ng/mL) showed AUCs of 0.87 and 0.89 with NPVs of 98%.

Conclusion: Neutrophil CD64, CD66b, and serum thioredoxin outperform conventional markers for detecting delayed FRI and, in combination, provide a high-negative predictive value panel that may substantially improve early diagnosis and safe exclusion of infection after fracture fixation.

## Introduction

Fracture-related infection (FRI) is a serious complication of operative fracture fixation, leading to delayed union or non-union, repeated surgeries, prolonged hospitalization, and substantial socioeconomic burden. Its true impact was long underestimated due to heterogeneous definitions, which prompted an international expert group to develop a consensus definition based on standardized clinical, radiological, and microbiological criteria [1]. Reported infection rates vary with fracture type and soft-tissue status, with approximately 1-2% in closed fractures and ≥8% in open fractures, especially in high-energy injuries [2]. With improving trauma survival and rising volumes of internal fixation, FRI is expected to represent a growing share of orthopedic complications [1,2].

Diagnosing FRI, particularly delayed forms, is difficult because early postoperative inflammatory responses to surgery mimic infection, and clinical signs may be subtle. Current diagnostic pathways, therefore, utilize composite major and minor criteria, wound breakdown, sinus tracts, purulence, culture results, and radiological changes within the FRI consensus framework [1,3]. However, these are often met only once infection is established, and cultures may be negative in low-grade or partially treated infections, leaving a diagnostic gap in the early phase [3]. In this study, the term "delayed FRI" refers to infection presenting in the delayed postoperative phase (two to 10 weeks after fixation), which is the operational temporal definition used for analysis and interpretation.

Conventional serum markers (total leucocyte count (TLC), erythrocyte sedimentation rate (ESR), and C-reactive protein (CRP)) are widely used but show substantial overlap between infected and non-infected patients and only modest diagnostic accuracy [3]. A recent review concluded that while they contribute to suggestive criteria, they are insufficient as stand-alone tests, particularly for delayed or low-grade FRI [4,5]. This underscores the need for more specific host-response biomarkers.

Neutrophil CD64 (FcγRI) is low at baseline but is rapidly upregulated by cytokines and bacterial products; a meta-analysis showed higher sensitivity and specificity for bacterial infection than traditional markers [6]. In musculoskeletal infection, CD64 performs comparably to CRP and procalcitonin in distinguishing septic from aseptic conditions, and neutrophil activation markers, including CD64 and CD66b, are strongly upregulated in systemic inflammation [7]. Thioredoxin, a key redox-active protein, is released during oxidative stress and tissue injury; in trauma populations, plasma thioredoxin rises sharply after injury and predicts post-injury sepsis and adverse outcomes [8]. Accordingly, we hypothesized a complementary diagnostic model in which CD64 and CD66b reflect neutrophil activation/degranulation, whereas thioredoxin reflects oxidative-stress biology, thereby capturing related but non-identical components of the host response to evolving FRI.

Overall, existing evidence shows that FRI is common and morbid, conventional markers are inadequate for early reliable diagnosis, and novel biomarkers reflecting neutrophil activation (CD64/CD66b) and oxidative stress (thioredoxin) are promising but untested in a large, prospective fracture cohort [3-8]. The primary objective of the present study was to compare the diagnostic accuracy of CD64, CD66b, and thioredoxin against conventional inflammatory markers using receiver operating characteristic (ROC) analysis, while secondary objectives were to describe perioperative biomarker trajectories and their correlations with TLC, ESR, and CRP. Early diagnosis was operationalized through sampling on postoperative days 3, 7, and 10.

## Materials and methods

The present investigation was a prospective cohort study conducted in the Department of Orthopedic Surgery and Biochemistry, King George's Medical University, Lucknow, between October 2020 and September 2022. Consecutive patients presenting over three years with acute long-bone fractures (upper or lower limb) requiring internal fixation were screened. The study received Institutional Ethics Committee approval (524/Ethics/2020; dated 19/06/2020) and adhered to the Declaration of Helsinki.

All skeletally immature and adult patients with acute long-bone fractures planned for internal fixation were considered eligible. The exclusion criteria were open fractures with gross contamination, polytrauma requiring prolonged ICU stay, pre-existing chronic osteomyelitis or prior surgery at the same fracture site, known immunodeficiency, chronic inflammatory or autoimmune disease, active systemic infection at the time of index surgery, malignancy, chronic kidney or liver failure, and long-term immunosuppressive therapy. Patients in whom informed consent could not be obtained, or adequate follow-up was not feasible, were also excluded.

**Figure 1:**
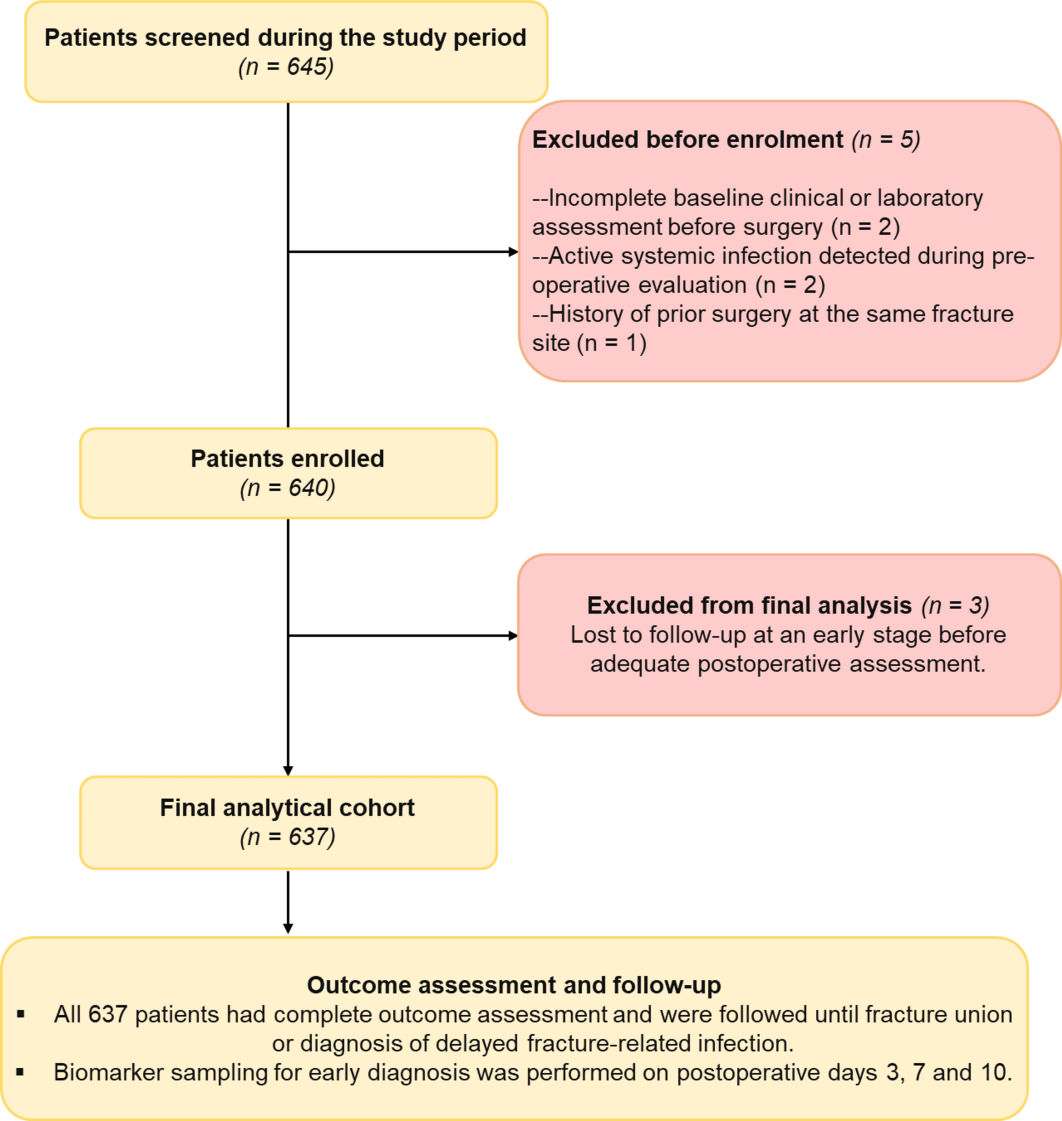
Patient recruitment, exclusions, and follow-up flow diagram

The minimum required sample size was calculated as 510 subjects, assuming an expected delayed FRI incidence of 8%, a 95% confidence level, 5% absolute precision, and a design effect of 1. During the study period, 645 patients were screened. Of these, five patients were excluded before enrollment due to genuine clinical or logistical reasons: incomplete baseline clinical or laboratory assessment before surgery (n=2), active systemic infection detected during preoperative evaluation (n=2), and a history of prior surgery at the same fracture site (n=1). Thus, 640 patients were enrolled. Among the enrolled patients, three were lost to follow-up at an early stage before adequate postoperative assessment and outcome adjudication could be completed; therefore, they were excluded from the final analysis. The final analytical cohort comprised 637 patients, all of whom had complete outcome assessment and were followed until fracture union or diagnosis of delayed FRI (Figure 1).

All patients underwent standard preoperative evaluation and routine investigations. Fractures were stabilized with internal fixation (nail, plate, or screw constructs) under regional or general anesthesia, according to surgeon preference. Perioperative antibiotic prophylaxis, soft-tissue handling, and postoperative care followed departmental protocols.

For biomarker assessment, venous blood was collected at four predefined time points: preoperatively (within 24 hours before surgery) and on postoperative days 3, 7, and 10. At each time point, 5-7 mL of venous blood was drawn under aseptic precautions and partitioned for hematological, biochemical, flow-cytometric, and enzyme-linked immunosorbent assay (ELISA)-based assays. Conventional inflammatory markers included TLC, ESR, and CRP. TLC was measured on an automated hematology analyzer; ESR was determined by the Wintrobe method within two hours of sampling; CRP was quantified using a high-sensitivity turbidimetric immunoassay on a fully automated clinical chemistry platform.

For immunophenotyping, ethylenediaminetetraacetic acid (EDTA)-anticoagulated whole blood was stained with fluorochrome-conjugated monoclonal antibodies to CD64 and CD66b following manufacturer instructions. After red cell lysis and washing, samples were acquired on a calibrated flow cytometer. Neutrophils were gated using forward/side scatter and CD45 expression, and the median fluorescence intensity (MFI) of CD64 and CD66b on neutrophils was recorded. Serum thioredoxin was measured using a commercial sandwich ELISA kit. Serum was separated by centrifugation and stored at -80 °C until batch analysis; all samples were run in duplicate, and mean values (ng/mL) were used. Internal controls and calibrators were included in each run.

Fracture severity was recorded using the Gustilo-Anderson classification for open fractures and the Tscherne-Oestern classification for soft-tissue injury in closed fractures [9,10]. Open fractures in the present cohort were limited to selected Gustilo grade II-III injuries without gross contamination, whereas grossly contaminated open fractures were excluded; this restriction is relevant when considering confounding by injury severity and the generalisability of biomarker performance.

Postoperatively, patients were followed clinically and radiologically at two weeks and subsequent visits according to departmental protocols. At each visit, the operated limb was examined for pain, warmth, erythema, swelling, wound discharge, sinus formation, and functional impairment. Radiographs were evaluated for alignment, implant position, signs of loosening, and bone lysis. Delayed FRI was operationally defined as FRI presenting in the delayed postoperative phase (two to 10 weeks after fixation) and was adjudicated using published consensus criteria incorporating clinical, radiological, and microbiological parameters. Patients with suspected infection (persistent pain, redness, delayed wound healing, discharge, or fever with raised inflammatory markers) underwent further work-up, repeat imaging, and surgical debridement when indicated. Microbiological sampling was therefore pursued in clinically suspected cases rather than systematically in all enrolled patients. In such cases, deep tissue and/or bone samples were obtained for Gram stain and quantitative aerobic and anaerobic culture using standard methods. Ultimately, patients were classified as non-FRI, clinically suspected culture-negative FRI, or culture-positive FRI. Inflammatory markers were treated as supportive/suggestive rather than confirmatory features; nevertheless, because they informed clinical suspicion and downstream work-up, a degree of incorporation bias is possible.

The primary outcome was the development of delayed FRI. Main exposures were perioperative levels and trajectories of CD64, CD66b, and thioredoxin, compared with TLC, ESR, and CRP. Continuous variables were summarized as mean ± standard deviation, and categorical variables as frequencies and percentages. Group comparisons used an independent-samples t-test for continuous variables and a chi-square test or Fisher's exact test for categorical variables. In the present study, serial biomarker temporal changes obtained at the predefined perioperative time points were analyzed in SPSS using repeated-measures ANOVA (General Linear Model, repeated-measures procedure) to examine within-subject changes over time and between-group differences between the FRI and non-FRI cohorts. However, we acknowledge that a formal assessment of the sphericity assumption was not performed. Correlations between novel and conventional markers were assessed using Pearson's or Spearman's coefficients. Diagnostic performance was evaluated by constructing ROC curves for each biomarker at each time point, using overall FRI status (including both clinically adjudicated culture-negative FRI and culture-positive FRI) as the reference. Comparative performance between biomarkers was assessed by comparing AUCs. Optimal cut-offs were data-derived from ROC analysis based on the best combined sensitivity and specificity; internal validation of these cut-offs was not performed, and no multivariable adjustment was applied in the diagnostic accuracy analyses. A two-sided p-value <0.05 was considered statistically significant. All statistical analyses were performed using IBM SPSS Statistics for Windows, Version 26 (Released 2018; IBM Corp., Armonk, New York, United States).

## Results

**Figure 2:**
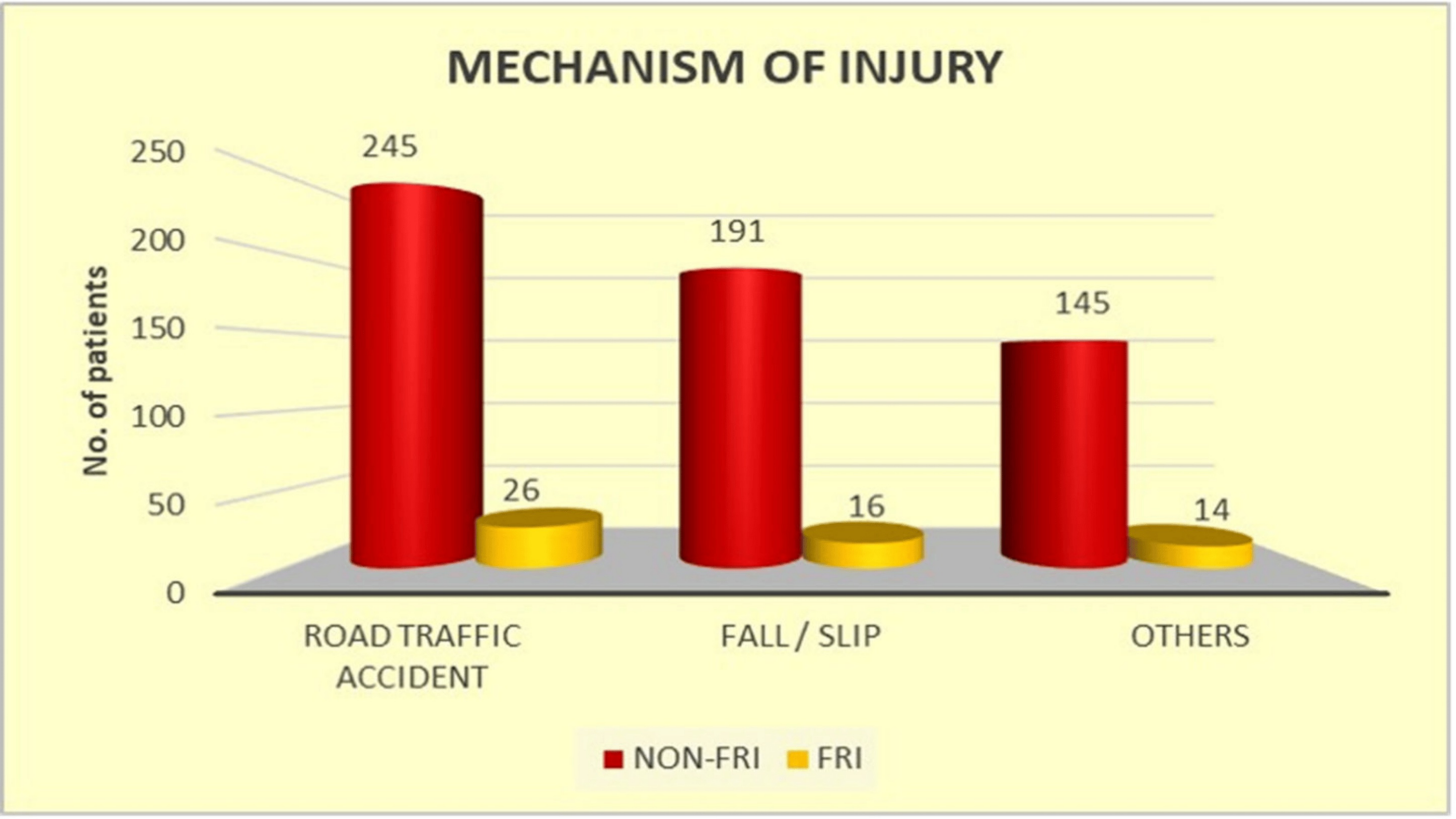
Graphical representation of the mechanism of injury of the study population FRI: fracture-related infection

A total of 637 patients with long-bone fractures managed by internal fixation were included in the final analysis, of whom 56 (8.8%) developed delayed FRI, and 581 (91.2%) had uneventful postoperative courses (Table 1). The mean age was comparable between the FRI and non-FRI groups (40.13 ± 13.44 vs 37.88 ± 12.61 years, p=0.2053), and there was no significant difference in sex distribution (male: 62.5% vs 54.9%, p=0.2747). The mechanism of injury was also similar, with road traffic accidents being the predominant cause in both groups (46.4% vs. 42.2%), followed by falls and other mechanisms (Table 1, Figure 2). The prevalence of diabetes mellitus and smoking/alcohol use and the proportion undergoing emergency fixation did not differ significantly between groups (all p>0.05) (Table 1). In contrast, markers of injury severity showed clear differences. Gustilo II-III open fractures were significantly more frequent in the FRI group than in the non-FRI group (30.4% vs 9.0%, χ²=24.23, p<0.0001), and a higher proportion of FRI patients had Tscherne grade ≥2 soft-tissue injury (78.6% vs 61.1%, χ²=6.661, p=0.0099) (Table 1).

**Table 1:** Baseline demographic and clinical characteristics of the study population (n=637) * denotes statistically significant values. FRI: fracture-related infection

Parameter	Non-FRI (n=581)	FRI (n=56)	Test (statistic) and p-value
Age (years)	37.88 ± 12.61	40.13 ± 13.44	t=1.268 p=0.2053
Gender			
Male	319 (54.9%)	35 (62.5%)	χ²=1.193 p=0.2747
Female	262 (45.1%)	21 (37.5%)	
Mechanism of injury			
Road traffic accident	245 (42.2%)	26 (46.4%)	χ²=0.508 p=0.7753
Fall/slip	191 (32.9%)	16 (28.6%)	
Others	145 (25.0%)	14 (25.0%)	
Open fracture (Gustilo II–III) [9]	52 (9.0%)	17 (30.4%)	X=24.23 p<0.0001*
Diabetes mellitus	81 (13.9%)	12 (21.4%)	χ²=2.296 p=0.1297
Smoking/alcohol use	209 (36.0%)	25 (44.6%)	χ²=1.652 p=0.1987
Emergency fixation	267 (46.0%)	32 (57.1%)	χ²=2.567 p=0.1091
Tscherne grade ≥ 2 [10]	355 (61.1%)	44 (78.6%)	χ²=6.661 p=0.0099
Time to union (weeks)	16.26 ± 3.42	21.84 ± 6.92	t=10.36 p<0.0001*

**Figure 3:**
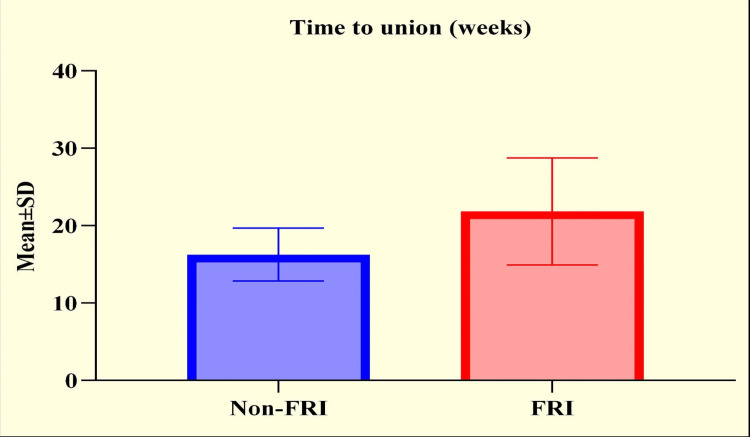
Graphical representation of time to union (weeks) of the study population FRI: fracture-related infection

Time to radiological union was markedly prolonged in patients with FRI (21.84 ± 6.92 weeks) compared with those without FRI (16.26 ± 3.42 weeks; t=10.36, p<0.0001) (Figure 3).

**Figure 4:**
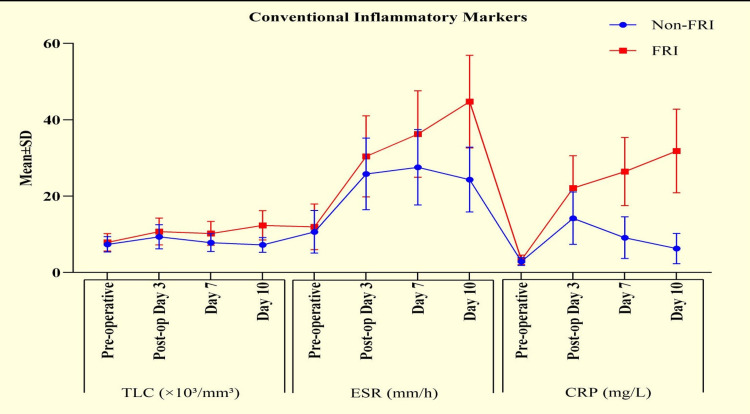
Graphical representations of comparison of conventional inflammatory markers between non-FRI and FRI at different time-points FRI: fracture-related infection; TLC: total leucocyte count; ESR: erythrocyte sedimentation rate; CRP: C-reactive protein

Conventional inflammatory markers showed no significant difference at baseline between the two groups for TLC and ESR, although preoperative CRP was slightly but significantly higher in the FRI group (3.31 ± 1.25 vs. 2.88 ± 1.03 mg/L; p=0.0036) (Table 2). From postoperative day 3 onwards, TLC, ESR, and CRP values diverged progressively, remaining elevated in the FRI group while declining in non-FRI patients. On day 3, TLC and ESR were already significantly higher in the FRI group (TLC: 10.73 ± 3.51 vs 9.35 ± 3.14 ×10³/mm³, p=0.0020; ESR: 30.42 ± 10.63 vs 25.82 ± 9.38 mm/h, p=0.0006). This difference widened further by days 7 and 10, when TLC, ESR, and CRP were all markedly raised in FRI patients (e.g., day 10 CRP: 31.83 ± 10.96 vs. 6.27 ± 3.96 mg/L, p<0.0001) (Table 2, Figure 4).

**Table 2:** Comparison of conventional inflammatory markers between non-FRI and FRI at different time-points * denotes statistically significant values. FRI: fracture-related infection; TLC: total leucocyte count; ESR: erythrocyte sedimentation rate; CRP: C-reactive protein

Marker / Time-point	Non-FRI (n=581)	FRI (n=56)	Test (statistic) and p-value
TLC (×10³/mm³)			
Preoperative	7.38 ± 2.03	7.89 ± 2.27	t=1.776 p=0.0762
Post-op Day 3	9.35 ± 3.14	10.73 ± 3.51	t=3.108 p=0.0020*
Day 7	7.83 ± 2.35	10.24 ± 3.12	t=7.099 p<0.0001*
Day 10	7.23 ± 1.93	12.35 ± 3.84	t=16.92 p<0.0001*
ESR (mm/h)			
Preoperative	10.65 ± 5.57	11.97 ± 5.96	t=1.683 p=0.0928
Post-op Day 3	25.82 ± 9.38	30.42 ± 10.63	t=3.462 p=0.0006*
Day 7	27.55 ± 9.87	36.25 ± 11.32	t=6.215 p<0.0001*
Day 10	24.33 ± 8.48	44.75 ± 12.12	t=16.48 p<0.0001*
CRP (mg/L)			
Preoperative	2.88 ± 1.03	3.31 ± 1.25	t=2.924 p=0.0036*
Post-op Day 3	14.22 ± 6.85	22.12 ± 8.44	t=8.063 p<0.0001*
Day 7	9.12 ± 5.44	26.43 ± 8.93	t=21.24 p<0.0001*
Day 10	6.27 ± 3.96	31.83 ± 10.96	t=36.74 p<0.0001*

**Figure 5:**
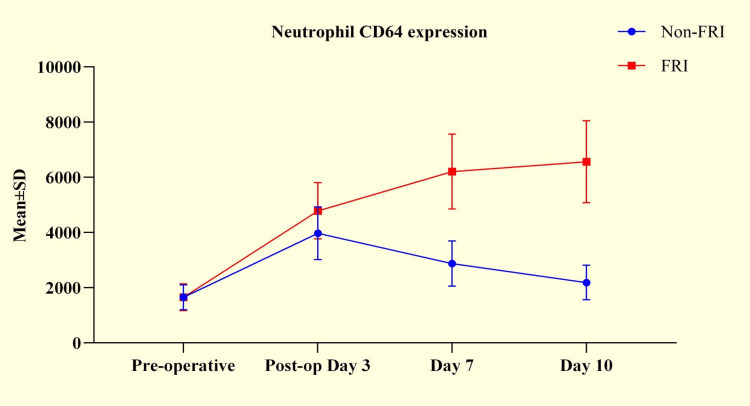
Graphical representations of the neutrophil CD64 expression between non-FRI and FRI at different time-points FRI: fracture-related infection

**Figure 6:**
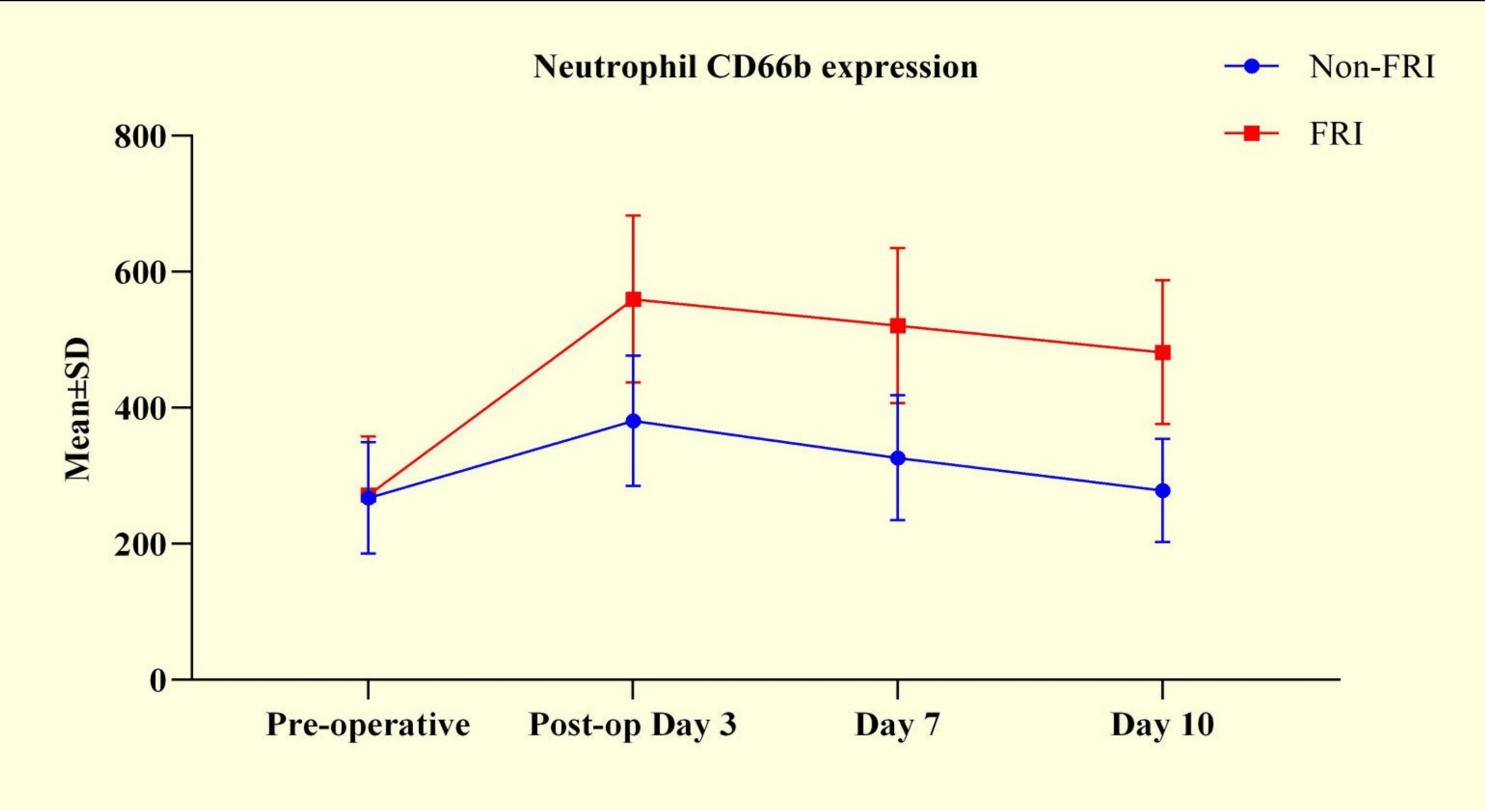
Graphical representations of neutrophil CD66b expression (median fluorescence intensity, MFI units) MFI: median fluorescence intensity; FRI: fracture-related infection

Neutrophil CD64 expression showed very similar baseline levels in both groups (preoperative MFI: 1653.81 ± 482.78 vs. 1652.67 ± 452.63; p=0.9857), but a clear divergence developed after surgery (Table 3, Figure 5). On day 3, CD64 MFI was significantly higher in the FRI group (4787.72 ± 1021.63 vs. 3973.63 ± 956.85; p<0.0001), and this gap increased markedly by day 7 (6208.78 ± 1356.86 vs. 2874.72 ± 814.75; p<0.0001) and day 10 (6563.95 ± 1482.87 vs. 2187.84 ± 623.63; p<0.0001) (Table 3). Neutrophil CD66b showed a similar pattern. Preoperative values were comparable (271.63 ± 85.96 vs. 267.63 ± 81.84; p=0.7281), but postoperative measurements were significantly higher in the FRI group at all time points. On day 3, CD66b MFI in the FRI group was 559.83 ± 122.74 versus 380.84 ± 95.85 in non-FRI patients (p<0.0001), with the difference persisting on days 7 (520.79 ± 113.85 vs. 326.63 ± 91.71; p<0.0001) and 10 (481.78 ± 105.86 vs. 278.41 ± 75.74; p<0.0001) (Table 3, Figure 6).

**Table 3:** Neutrophil CD64 expression (median fluorescence intensity, MFI units) * denotes statistically significant values. MFI: median fluorescence intensity; FRI: fracture-related infection

Time-point	Non-FRI (n=581)	FRI (n=56)	Test (statistic) and p-value
Neutrophil CD64 expression			
Preoperative	1652.67 ± 452.63	1653.81 ± 482.78	t=0.00178 p=0.9857
Post-op Day 3	3973.63 ± 956.85	4787.72 ± 1021.63	t=6.044 p<0.0001*
Day 7	2874.72 ± 814.75	6208.78 ± 1356.86	t=27.23 p<0.0001*
Day 10	2187.84 ± 623.63	6563.95 ± 1482.87	t=42.34 p<0.0001*
Neutrophil CD66b expression			
Preoperative	267.63 ± 81.84	271.63 ± 85.96	t=0.3478 p=0.7281
Post-op Day 3	380.84 ± 95.85	559.83 ± 122.74	t=12.99 p<0.0001*
Day 7	326.63 ± 91.71	520.79 ± 113.85	t=14.79 p<0.0001*
Day 10	278.41 ± 75.74	481.78 ± 105.86	t=18.44 p<0.0001*

**Figure 7:**
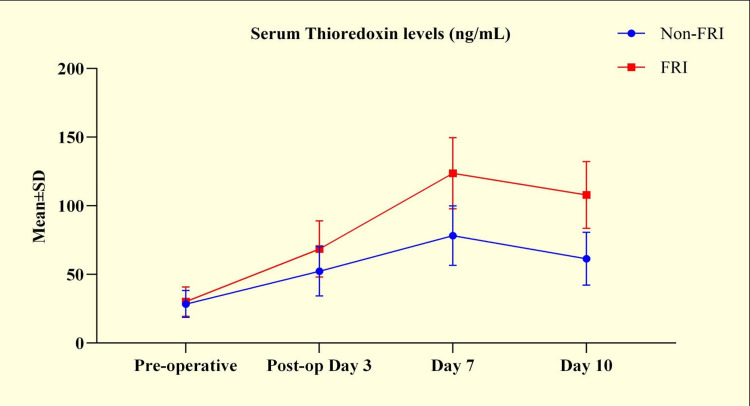
Graphical representations of serum thioredoxin levels (ng/mL) between non-FRI and FRI at different time-points FRI: fracture-related infection

Serum thioredoxin levels followed a comparable trajectory. Baseline values did not differ significantly (30.16 ± 10.73 vs. 28.48 ± 9.82 ng/mL; p=0.2258), but FRI patients exhibited substantially higher thioredoxin concentrations postoperatively. On day 3, thioredoxin levels were 68.52 ± 20.44 ng/mL in the FRI group compared with 52.31 ± 17.95 ng/mL in the non-FRI group (p<0.0001), with further divergence on day 7 (123.71 ± 25.86 vs. 78.25 ± 21.67 ng/mL; p<0.0001) and day 10 (107.92 ± 24.33 vs. 61.42 ± 19.24 ng/mL; p<0.0001) (Table 4, Figure 7).

**Table 4:** Serum thioredoxin levels (ng/mL) at different time-points in non-FRI and FRI groups * denotes statistically significant values. FRI: fracture-related infection

Time-point	Non-FRI (n=581)	FRI (n=56)	Test (statistic) and p-value
Preoperative	28.48 ± 9.82	30.16 ± 10.73	t=1.213 p=0.2258
Post-op Day 3	52.31 ± 17.95	68.52 ± 20.44	t=6.373 p<0.0001*
Day 7	78.25 ± 21.67	123.71 ± 25.86	t=14.72 p<0.0001*
Day 10	61.42 ± 19.24	107.92 ± 24.33	t=16.84 p<0.0001*

Correlation analysis at day 7 demonstrated that all three novel biomarkers were significantly associated with conventional inflammatory markers (Table 5). CD64 showed strong positive correlations with TLC (r=0.61, p<0.001) and CRP (r=0.68, p<0.001), and a moderate correlation with ESR (r=0.37, p<0.001). CD66b was similarly correlated with TLC (r=0.55, p<0.001) and CRP (r=0.59, p<0.001), with a weaker but significant association with ESR (r=0.31, p=0.002). Thioredoxin demonstrated moderate correlations with TLC (r=0.49, p<0.001), ESR (r=0.29, p=0.004), and CRP (r=0.52, p<0.001) (Table 5).

**Table 5:** Correlation between novel biomarkers and conventional inflammatory markers at day 7 (n=637) Pearson’s correlation coefficient (r) is shown with p-values in parentheses. * denotes statistically significant values. TLC: total leucocyte count; ESR: erythrocyte sedimentation rate; CRP: C-reactive protein

Biomarker (Day 7)	TLC (×10³/mm³)	ESR (mm/h)	CRP (mg/L)
CD64	0.61 (<0.001*)	0.37 (<0.001*)	0.68 (<0.001*)
CD66b	0.55 (<0.001*)	0.31 (0.002*)	0.59 (<0.001*)
Thioredoxin (ng/mL)	0.49 (<0.001*)	0.29 (0.004*)	0.52 (<0.001*)

ROC analysis highlighted clear differences in diagnostic performance between conventional and novel biomarkers. Among traditional markers, CRP on day 7 performed best, with an optimal cut-off of >20 mg/L yielding sensitivity 83%, specificity 80%, PPV 33%, NPV 98%, and an AUC of 0.85 (95% CI: 0.77-0.92). TLC on day 10 and ESR on day 10 had lower AUCs of 0.79 and 0.73, respectively, and similarly low PPVs (26% and 20%). In contrast, all three novel biomarkers achieved higher AUCs and excellent NPVs. CD64 on day 10, at a cut-off >3800 MFI, had sensitivity 91%, specificity 87%, PPV 41%, NPV 99%, and the highest AUC of 0.93 (95% CI: 0.87-0.98). CD66b on day 3 (cut-off >450 MFI) showed sensitivity 82%, specificity 81%, PPV 31%, and NPV 98% with an AUC of 0.87 (95% CI: 0.79-0.94), indicating strong early discriminatory capacity. Thioredoxin on day 7 (cut-off >85 ng/mL) yielded sensitivity 86%, specificity 83%, PPV 34%, NPV 98%, and an AUC of 0.89 (95% CI: 0.82-0.95) (Table 6).

**Table 6:** Diagnostic performance of biomarkers for delayed fracture-related infection (FRI vs. non-FRI) Diagnostic performance derived from receiver operating characteristic (ROC) analysis. FRI: fracture-related infection; TLC: total leucocyte count; ESR: erythrocyte sedimentation rate; CRP: C-reactive protein; PPV: positive predictive value; NPV: negative predictive value

Biomarker (best time-point)	Optimal cut-off	Sensitivity (%)	Specificity (%)	PPV (%)	NPV (%)	AUC (95% CI)
TLC (Day 10)	> 10.5 ×10³/mm³	74	78	26	97	0.79 (0.70–0.87)
ESR (Day 10)	> 35 mm/h	65	71	20	95	0.73 (0.63–0.82)
CRP (Day 7)	> 20 mg/L	83	80	33	98	0.85 (0.77–0.92)
CD64 (Day 10)	> 3 800 MFI	91	87	41	99	0.93 (0.87–0.98)
CD66b (Day 3)	> 450 MFI	82	81	31	98	0.87 (0.79–0.94)
Thioredoxin (Day 7)	> 85 ng/mL	86	83	34	98	0.89 (0.82–0.95)

Overall, these results demonstrate that while conventional markers reliably reflect systemic inflammation, CD64, CD66b, and thioredoxin provide superior diagnostic discrimination and very high negative predictive values for delayed FRI when assessed at their optimal postoperative time-points.

## Discussion

In this prospective cohort of 637 long-bone fractures treated with internal fixation, the overall incidence of delayed FRI was 8.8% (56/637), which lies at the upper end of reported rates for mixed cohorts of closed and selected open fractures in tertiary trauma settings. Large systematic reviews and narrative overviews report infection rates of roughly 1-2% after internal fixation of closed fractures and up to 20-30% following open fractures, particularly in high-energy injuries and severely contaminated wounds [11-14]. Our findings, therefore, align with the international literature and reflect the case mix of a high-volume trauma center managing a substantial proportion of complex injuries.

Open injury and soft-tissue severity emerged as major risk factors in our cohort. Gustilo II-III open fractures were significantly over-represented among FRI patients (30.4% vs 9.0%, p<0.0001), and a higher proportion of FRI cases had Tscherne grade ≥2 soft-tissue injury (78.6% vs 61.1%, p=0.0099). This mirrors systematic reviews of FRI risk factors, where open fractures and high-energy mechanisms are among the most robust predictors of infection and treatment failure [11-14]. At the same time, these findings indicate that fracture severity and soft-tissue injury are important potential confounders of perioperative biomarker trajectories, and biomarker elevations in some patients may partly reflect injury burden rather than infection per se.

Serial monitoring of TLC, ESR, and CRP revealed a pattern consistent with the recognized limitations of these markers in FRI. By day 10, AUCs for TLC, ESR, and CRP were 0.79, 0.73, and 0.85, respectively, but positive predictive values remained low despite high NPVs. These findings align with studies showing that conventional serum markers, although sensitive to systemic inflammation, lack specificity and pre-test discrimination in FRI [3-5].

Neutrophil CD64 was the best-performing biomarker in this study. On day 10, a cut-off of >3800 MFI yielded sensitivity 91%, specificity 87%, PPV 41%, and NPV 99%, clearly outperforming TLC, ESR, and CRP. These findings are concordant with prior literature on CD64 in bacterial and musculoskeletal infection [6,15-19]. CD66b also performed well and showed earlier separation by day 3, suggesting that it may provide early warning of an exaggerated innate immune response at the fracture site. Serum thioredoxin demonstrated strong discrimination at day 7, extending earlier observations on oxidative-stress biology in trauma and sepsis to FRI [8,20]. However, mechanistic interpretation should remain cautious because oxidative stress may also be elevated in severe trauma independent of infection.

Considering all biomarkers together, our study supports the view that reliance on conventional serum markers alone is inadequate for early FRI diagnosis [3,5]. In our cohort, CRP at day 7 was the best classical marker (AUC 0.85), yet still inferior to CD64 (AUC 0.93) and thioredoxin (AUC 0.89). Importantly, CD64, CD66b, and thioredoxin all showed very high NPVs (98-99%), suggesting that a combined biomarker panel could be valuable for ruling out FRI in patients with nonspecific pain or mild biochemical abnormalities during the subacute postoperative period. Because the novel markers showed moderate correlations with CRP and TLC rather than complete redundancy, they may offer complementary information; however, additive diagnostic value beyond conventional markers was not formally tested in multivariable models in the present study.

The strengths of this study include its large sample size, prospective design, predefined perioperative sampling, and use of contemporary FRI consensus criteria. Several limitations warrant emphasis. First, microbiological confirmation was pursued only in clinically suspected cases, introducing possible verification bias. Second, because inflammatory markers contributed to clinical suspicion and downstream work-up, some incorporation bias is possible. Third, diagnostic cut-offs were derived from this cohort without internal validation, so optimism and overfitting cannot be excluded. Fourth, although serial biomarker measurements were analyzed longitudinally using repeated-measures procedures, formal testing of assumptions underlying this approach, including Mauchly’s test of sphericity, was not undertaken; accordingly, the robustness of within-subject time-point comparisons may be limited. Fifth, no multivariable adjustment was performed to account for confounding by open fracture status and soft-tissue severity. Sixth, the exclusion of grossly contaminated open fractures and ICU polytrauma means that the cohort represents a somewhat lower-risk spectrum, which may limit generalisability to more severe trauma populations. Finally, the single-center design and fixed sampling times may limit broader applicability.

In summary, this prospective cohort of 637 patients showed that delayed FRI occurred in about 9% and was clearly associated with open fractures, higher Tscherne soft-tissue grades, and significantly prolonged time to union. Conventional inflammatory markers diverged postoperatively between FRI and non-FRI groups but were limited by modest specificity and low PPVs. In contrast, neutrophil CD64, CD66b, and serum thioredoxin demonstrated superior diagnostic accuracy, with higher AUCs and excellent NPVs (≥98%). CD64 on day 10 was the best single discriminator, CD66b provided earlier separation by day 3, and thioredoxin performed optimally at day 7, reflecting complementary aspects of neutrophil activation and oxidative stress. These findings support the potential role of a CD64-CD66b-thioredoxin panel as an adjunct to clinical and radiological assessment, but external validation in independent multicenter cohorts is required before clinical implementation.

## Conclusions

In this cohort, FRI occurred in 8.8% (56/637) of patients. Conventional inflammatory markers showed limited discriminatory value, with mean CRP and TLC failing to reliably differentiate FRI from non-FRI cases, while erythrocyte sedimentation rate demonstrated a statistically significant association with infection.

In contrast, neutrophil CD64, CD66b, and thioredoxin levels were significantly higher in patients with FRI, supporting their potential role as adjunctive biomarkers for earlier identification of FRI. These findings should be interpreted as promising but preliminary and require validation in independent cohorts before routine clinical use.
